# The bibliometric analysis of studies on intracytoplasmic sperm injection from 2002 to 2021

**DOI:** 10.3389/fendo.2023.1109623

**Published:** 2023-03-14

**Authors:** Xiaoli Shen, Tianbing Xiao, Wei Han, Hong Ye, Yuanfeng Zhang, Guoning Huang

**Affiliations:** ^1^ Center for Reproductive Medicine, Chongqing Key Laboratory of Human Embryo Engineering, Chongqing Health Center for Women and Children, Women and Children’s Hospital of Chongqing Medical University Chongqing, Chongqing, China; ^2^ Department of Urology, People’s Hospital of Fengjie, Chongqing, China; ^3^ Department of Urology, The Second Affiliated Hospital of Chongqing Medical University, Chongqing, China

**Keywords:** intracytoplasmic sperm injection (ICSI), bibliometric analysis, infertility, research hotspots, data visualization, assisted reproductive technology

## Abstract

**Background:**

Infertility is estimated to occur in 1 out of every 4–7 couples. Intracytoplasmic sperm injection (ICSI), a type of assisted reproduction introduced in 1992, has been used across the world for almost all indications of infertility, yielding high pregnancy rates. There is a growing concern worldwide about ICSI since semen quality has declined in recent years, accompanied with the potential risks of this technology. This study aims to analyze the current status and hotspots of ICSI *via* a bibliometric analysis.

**Methods:**

We retrieved publications on ICSI from the Web of Science Core Collection database from 2002 to 2021. CiteSpace was used to summarize knowledge mapping of subject categories, keywords, and co-citation relationships with the strongest citation bursts. VOSviewer was used to explore co-citation and co-occurrence relationships for countries, organizations, references, authors, and keywords.

**Results:**

A total of 8271 publications were analyzed between 2002 and 2021. The major findings are as follows: the USA, China, Italy, Japan, and Belgium are the top five prolific countries. The Free University of Brussels, University of Copenhagen, University of Valencia, Ghent University, and the University of California San Francisco are the top five contributing organizations. *Fertility and Sterility* and *Human Reproduction* are the most productive and cited journals. The hotspot topics are risks of ICSI, oocyte preservation, live birth rate, infertile men, and embryo quality in the past two decades.

**Conclusion:**

This study presents a research overview of ICSI from different perspectives. These findings will contribute to a better understanding of the current status of ICSI research and provide hotspots and trends for future studies.

## Introduction

Infertility is defined as failure to achieve a clinical pregnancy after one year of unprotected sexual intercourse. It is estimated that 8% to 12% of reproductive-aged couples are affected by infertility worldwide (1, 2), with male problems accounting for approximately 50% of sterility cases (3, 4). Assisted reproductive technology (ART) includes all fertility treatments in which either eggs or embryos are manipulated to accomplish successful pregnancy. In general, ART comprises *in vitro* fertilization (IVF) and intracytoplasmic sperm injection (ICSI). ICSI involves a single sperm being microinjected into an oocyte. It has become increasingly employed as a form of infertility therapy. Compared to traditional IVF, ICSI appears to be more successful in helping infertile men to be parents. ICSI is usually performed when the sperm is unable to fertilize the egg. Only about 5% of infertile men could be treated without ICSI (5). Therefore, ICSI restores the fertilization rate to normal in couples with unexpectedly low fertilization rates due to a decline in semen quality. However, issues regarding the health of offspring obtained *via* ICSI have been discussed for years. There are minor potential risks of congenital and epigenetic disorders in infants who were conceived through ICSI (6, 7). Given the importance of the sperm epigenome to early embryogenesis, ICSI technology might increase the frequency of imprinting disorders and change epigenetic reprogramming, eventually adversely affect embryo evolution and long-life health of offspring by using immature spermatozoa that may not have been adequately imprinted or methylated (8, 9). Some evidences demonstrated the severity of male factor infertility does not seem to impact on cognitive development in early childhood among children conceived with ICSI, while many studies are limited by small sample size and the potential for ICSI by itself to influence cognitive outcomes requires elucidation (10, 11). During the last two decades, substantial progress has been made in ART research. Although the number of ICSI procedures has been increasing year by year, the associated birth defects are still not only one of the frontier problems but also the focus of research (12).

Bibliometric analysis was used to summarize qualitative and quantitative contributions in a specific research field; for instance, the numbers of citations and publications of authors, journals, countries, institutions, keywords, and influential articles (13). Furthermore, a bibliometric analysis could also reflect research hotspots and study trends through keyword and item analyses (14). To date, there is still no bibliometric study that illustrates hotspots and summarizes the research status in the ICSI field during the past 20 years.

This study aimed to quantitatively explore the publishing trend, authors, institutions, cited articles, countries, journals, and keywords with the strongest citation burst detection in the field of ICSI research. The resultant analysis should characterize the insight into the most influential articles in the field of ICSI. This study also attempted to identify potential future hotspots and trends in this area *via* the overlay map of co-occurring keywords and clustering visualization analyses.

## Materials and methods

### Data sources and search strategy

The Web of Science Core Collection (WoSCC) database was used as the data source. The citation indexes include ‘Science Citation Index Expanded,’ ‘Current Chemical Reactions,’ and ‘Index Chemicus’. Study data were retrieved by using the following index terms during the 2002–2021 period: TS = (“Intracytoplasmic Sperm Injection” or “ICSI”). The data source was extracted on September 28, 2022. The publication type included articles and reviews but excluded meeting abstracts, letters, reports, news, etc. The language was limited to English. The search results were exported with “full records and full contents” stored in “txt” format as the input source for CiteSpace and VOSviewer. The database was searched and screened independently by W. Han and Y. Zhang. Differences in viewpoints between these two were resolved *via* discussions with the whole author team until a consensus was reached. In the end, a total of 8271 publications were extracted for analysis.

### Data extraction and analytical methods

Several bibliometric analysis software packages with different capabilities and limitations have been used; for example, Perish, CiteSpace, HistCite, BibExcel, and VOSviewer. For the objectivity of ICSI research results, we used CiteSpace 6.1.R3, VOSviewer 1.6.18, and an online platform of literature metrology analysis (https://bibliometric.com/) to perform bibliometric analyses (15, 16).

CiteSpace was used to analyze the keywords and trends in this research area. We used VOSviewer to visualize the number of publications, identify those with the most citations, and classify publications by author, organization, country, and journal. Next, we summarized the number of citations, article title, first author’s name, year, journal of publication, and country of the corresponding author using Microsoft Excel 2010 and online platforms for literature metrology analysis. The 2021 impact factor (IF) of each journal was extracted from the latest Journal Citation Reports (JCR, 2021) using Clarivate Analytics. The keyword bursts were used to identify the hotspots and trends in the field of ICSI research. In the visualization of CiteSpace and VOSviewer, the node size represents the number of publications while the line thickness indicates the strength of the relationship. The parameters of CiteSpace were set as follows: time slicing (2002–2021), years per slice ‘3’, term source (all selection), and visualization (cluster view/time zone view). The main steps for setting up a bibliometric analysis were operated as follows (1): importing the publications and formatting the data (2), restricting the term sources (3), setting the selection criteria or the minimum number of co-occurrence and co-citations.

## Results

### General study data

**Figure 1:**
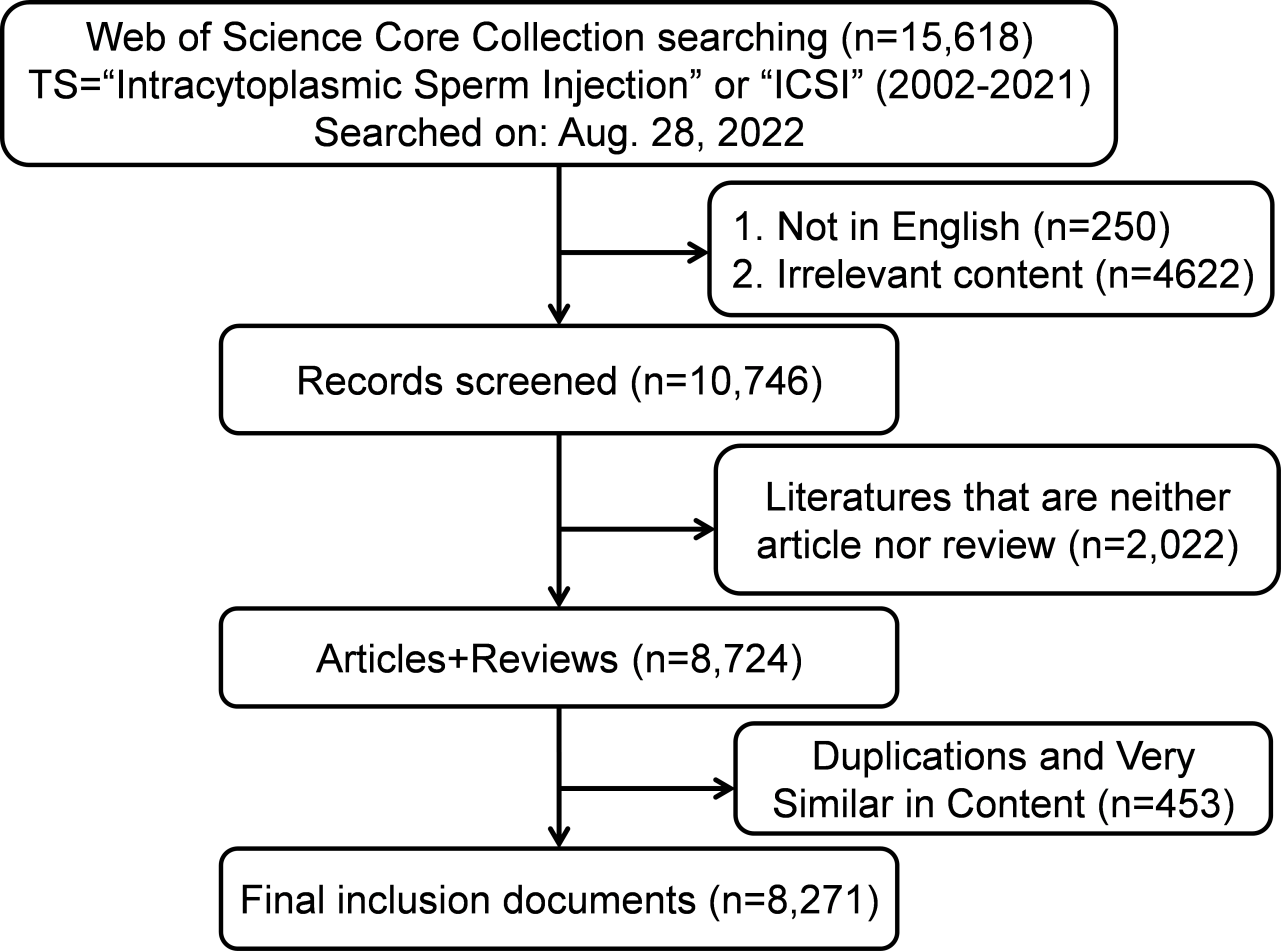
Flow chart of data screening and bibliometric analysis on ICSI research.

Based on the screening criteria, a total of 15,618 publications were identified using the index terms “intracytoplasmic sperm injection” or “ICSI” from January 1, 2002, to December 31, 2021. After excluding non-English manuscripts (n=250) and the irrelevant publications (n=4622), there were 10746 publications. Duplicate publications were excluded by CiteSpace. Finally, 8271 publications (7081 articles and 1190 reviews) were selected for analysis per the above screening conditions (**Figure 1**). A total of 5932 of these publications were cited more than 10 times.

**Figure 2:**
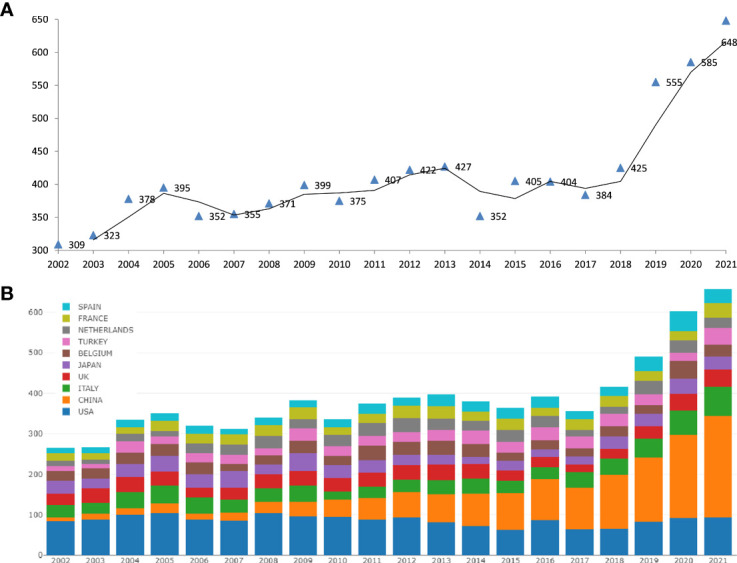
The number of ICSI research publications indexed by the WoSCC from 2002 to 2021. Distribution trends of publications by year **(A)** and country **(B)**.

The number of ICSI research publications increased from 309 in 2002 to 648 in 2021 as indexed by the WoSCC (**Figure 2A**). The rate of production of publications grew fast between 2018 and 2021. The top fifteen most cited publications ranged from 458 to 1337 citations as shown in **Table 1**. The most cited article was a recommendation published in 2006 in *Journal of Clinical Oncology* by Stephanie J. Lee et al. from the American Society of Clinical Oncology, with 1337 citations (17).

**Table 1:** Top 15 high-cited references in the field of ICSI research from 2002-2021.

First Author	TC	Journal	Title	Institution	Year
Lee, Stephanie J	1337	Journal of Clinical Oncology	American Society of Clinical Oncology recommendations on fertility preservation in cancer patients	Cornell University	2006
Kuwayama, M	878	Reproductive Biomedicine Online	Highly efficient vitrification method for cryopreservation of human oocytes	Aarhus University	2005
Jackson, RA	818	Obstetrics & Gynecology	Perinatal outcomes in singletons following *in vitro* fertilization: A meta-analysis	University of California San Francisco	2004
Broekmans, F. J.	781	Human Reproduction Update	A systematic review of tests predicting ovarian reserve and IVF outcome	Vrije University Amsterdam	2006
Hansen, M	762	New England Journal of Medicine	The risk of major birth defects after intracytoplasmic sperm injection and *in vitro* fertilization.	University of Leicester	2002
DeBaun, MR	674	American Journal of Human Genetics	Association of *in vitro* fertilization with Beckwith-Wiedemann syndrome and epigenetic alterations of LIT1 and H19	Johns Hopkins University	2003
Inhorn, Marcia C	623	Human Reproduction Update	Infertility around the globe: new thinking on gender, reproductive technologies and global movements in the 21st century	Yale University	2015
Lanfranco, F	575	Lancet	Klinefelter’s syndrome	University of Munster	2004
AmerSocReprod Med	553	Fertility and Sterility	Mature oocyte cryopreservation: a guideline	AmerSocReprod Med	2013
Wallace, WHB	537	Lancet Oncology	Fertility preservation for young patients with cancer: who is at risk and what can be offered?	University of Edinburgh	2005
Meseguer, Marcos	529	Human Reproduction	The use of morphokinetics as a predictor of embryo implantation	University of Valencia	2011
Cox, GF	518	American Journal of Human Genetics	Intracytoplasmic sperm injection may increase the risk of imprinting defects	Harvard University	2002
Davies, MJ	514	New England Journal of Medicine	Reproductive Technologies and the Risk of Birth Defects	University of Adelaide	2012
Bungum, M	458	Human Reproduction	Sperm DNA integrity assessment in prediction of assisted reproduction technology outcome	Lund University	2007
Hansen, M	458	Human Reproduction	Assisted reproductive technologies and the risk of birth defects - a systematic review	University of Western Australia	2005

TC, total citations.

### Distribution and contribution of countries

**Figure 3:**
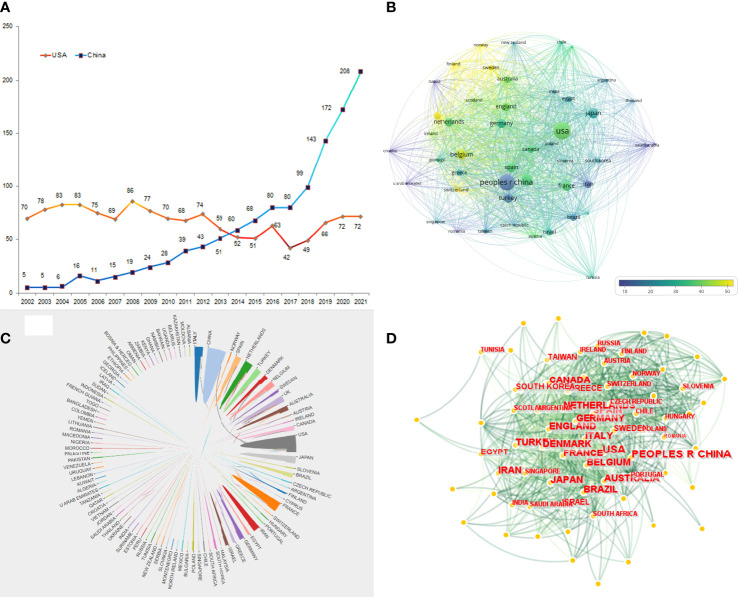
Distribution of country-specific studies in the dataset; Trends of publications between China and USA were compared during past two decades **(A)**. The co-occurrence of citations among different countries was visualized from VOSviewer **(B)** and CiteSpace **(D)**. The cooperation network of different regions was drawn by online bibliometric analysis **(C)**. Node size indicates the number of publications.

The United States accounted for 22.65% of all publications (n=309) in 2002. In the early stages, the United States, Belgium, Japan, Italy, and the UK contributed the majority of studies on ICSI, both in terms of number and citation rate. After 2018, these parameters increased rapidly in China, the UK, Italy, and Spain, with China accounting for 32.10% of all publications on ICSI from 2018 to 2021. All these results are presented in **Figure 2A** and **Figure 2B**. Next, we compared the two countries with the highest numbers of publications; i.e., the United States and China. The number of studies conducted in the United States has remained fairly constant in the past 20 years. However, China grew exponentially from only five publications in 2002 to 208 publications in 2021 as shown in **Figure 3A**. Consequently, we analyzed the collaboration between different countries using VOSviewer, CiteSpace, and online resources. The results show that the USA, China, Japan, and Belgium collaborate closely in the field of ICSI research (**Figures 3B–D**).

### Contribution of institutions

**Figure 4:**
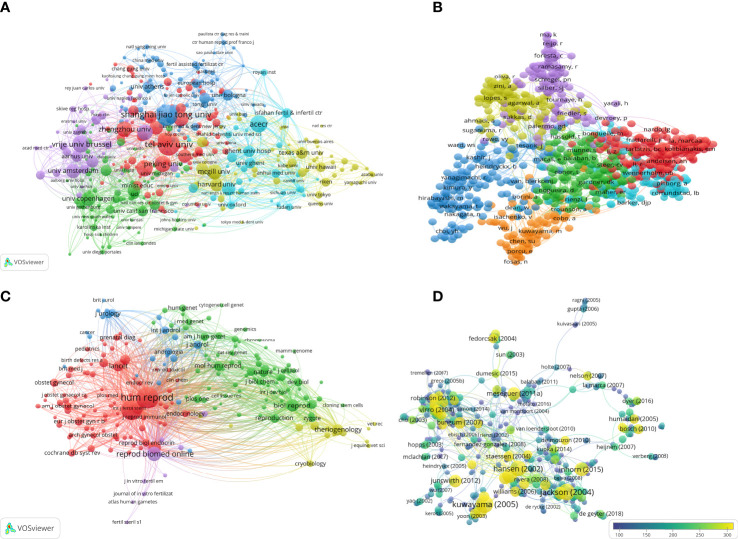
The co-citation map and cooperation network of institutions **(A)**, authors **(B)** and journals **(C)** based on VOSviewer analysis. Node size indicates the number of publications **(A, C)**. The most cited publications were visualized by VOSviewer **(D)**, different color means the range of citation times. The higher the centrality of a node, the more influential and important it is **(C, D)**.

There are many institutions that contribute constantly to the ICSI field. Institutional contributions may vary, as the CiteSpace and VOSviewer algorithms include the addresses of all authors that appear in the same articles. Some articles appeared in two related units at the same time, such as the University of Copenhagen and Copenhagen University Hospital, or different languages of the same correspondence address, for example, the Free University of Brussels apnd *Vrije Universiteit Brussel*. These discrepancies could make the contributions of the same unit discrete. To solve this problem, we united similar addresses to a consolidated institution. The Free University of Brussels had a total of 23547 citations in the past 20 years, the average number of citations of each paper (ACP) was 97.71. The University of California San Francisco was the most cited organization in the North American area, as it was cited 3071 times and its ACP was 63.98 in the field of ICSI research. The Free University of Brussels, University of Copenhagen, University of Valencia, and Ghent University had the most citations in ICSI research in Europe (**Table 2**). The Shanghai Jiao Tong University had the most citations among Asian countries, with a total of 2366 citations; however, the ACP was 20.05. The Free University of Brussels was the most productive institution, with 241 manuscripts published in the past two decades, followed by the University of Copenhagen (n=146), Ghent University (n=125), Tel Aviv University (124), and Shanghai Jiao Tong University (n=118); these are the top five institutions (**Table 3**). The network knowledge map among institutions was generated using VOSviewer as shown in **Figure 4A**.

**Table 2:** Top 15 most cited affiliations in the field of ICSI research.

Rank	Affiliation	Documents	Citations	TLS	ACP
1	Free University of Brussels	241	23547	4930	97.71
2	University of Copenhagen	146	8043	3246	55.09
3	University of Valencia	101	5423	1957	53.69
4	Ghent University	125	3719	2845	29.75
5	University of California San Francisco	48	3071	1034	63.98
6	University of Pennsylvania	35	2961	703	84.60
7	Harvard University	64	2813	956	43.95
8	University of Adelaide	52	2631	985	50.60
9	Yale University	49	2549	859	52.02
10	Tel Aviv University	124	2476	1243	19.97
11	ESHRE Central Office	18	2449	638	136.06
12	Shanghai Jiao Tong University	118	2366	1236	20.05
13	University of Bologna	52	2352	846	45.23
14	European Hospital	38	2278	974	59.95
15	University of Helsinki	38	2245	711	59.08

ACP, average citations per publication; TLS, total link strength.

**Table 3:** Top 15 most prolific affiliations in terms of ICSI research.

Rank	Affiliation	Documents	Citations	TLS	ACP
1	Free University of Brussels	241	23547	4930	97.71
2	University Of Copenhagen	146	8043	3246	55.09
3	Ghent University	125	3719	2845	29.75
4	Tel Aviv University	124	2476	1243	19.97
5	Shanghai Jiao Tong University	118	2366	1236	20.05
6	University of Valencia	101	5423	1957	53.69
7	Academic Center for Education, Culture and Research (ACECR)	91	1176	1219	12.92
8	Peking University	85	1162	880	13.67
9	Shandong University	85	1096	706	12.89
10	Zhengzhou University	78	585	753	7.50
11	Sun YatSen University	77	1203	640	15.62
12	Mcgill University	74	2193	814	29.64
13	Cairo University	74	1313	489	17.74
14	University of Amsterdam	70	1730	971	24.71
15	Tehran University of Medical Sciences	70	961	422	13.73

ACP, average citations per publication; TLS, total link strength.

### Top contributing authors

Knowledge mapping provides information on potential productive and influential scholars. The top three authors with the largest number of publications were Tournaye Herman (n=97), Devroey Paul (n=92), and De Sutter Petra (n=69). The details of the most productive authors are shown in **Table 4**. On the other hand, the authors with the most citations were Devroey Paul from the Free University of Brussels with 6358 total citations and an average of 69.11 citations per paper, followed by Van Steirteghem A, also from the Free University of Brussels, with 4873 total citations and an average of 85.49 citations per publication. The top 15 most cited authors are summarized in **Table 5**. The authors’ cooperation network was visualized by VOSviewer. The size of a node represents the number of publications and different colors represent the authors of a given cluster. Further network mapping was presented by VOSviewer to identify the co-citation related to ICSI research in the last two decades. These results show the most active and fruitful authors in the field of ICSI research (**Figure 4B**).

**Table 4:** Top 15 most productive authors in the field of ICSI research.

Author	Documents	Citations		TLS	Organizations
Tournaye, Herman	97	4271		93	Free University of Brussels
Devroey, Paul	92	6358		91	Free University of Brussels
De Sutter, Petra	69	2426		60	Ghent University
Borges, Edson, Jr.	60	1118		59	Fertility Medical Group & Sapientiae Institute, Sao Paulo
Andersen, Anders Nyboe	59	4832		50	University of Copenhagen
Van Steirteghem, A	57	4873		55	Free University of Brussels
Iaconelli, Assumpto, Jr.	56	1026		56	Fertility Medical Group & Sapientiae Institute, Sao Paulo
Pellicer, Antonio	46	2464		40	University of Valencia
Setti, Amanda Souza	46	674		26	Fertility Medical Group & Sapientiae Institute, Sao Paulo
Kuang, Yanping	44	1183		42	Shanghai Jiao Tong University
Qiao, Jie	44	557		42	Peking University
Remohi, Jose	43	3061		43	University of Valencia
De Almeida Ferreira Braga, Daniela Paes	40	687		40	Fertility Medical Group & Sapientiae Institute, Sao Paulo
Meseguer, Marcos	40	2829		36	University of Valencia
Wakayama, Teruhiko	37	1040		34	University of Hawaii System

TLS, total link strength.

**Table 5:** Top 15 most influential authors involved in ICSI research.

Author	Documents	Citations	TLS	Organizations
Devroey, Paul	92	6358	91	Free University of Brussels
Van Steirteghem, A	57	4873	55	Free University of Brussels
Andersen, Anders Nyboe	59	4832	50	University of Copenhagen
Tournaye, Herman	97	4271	93	Free University of Brussels
De Mouzon, Jacques.	26	3136	25	National Institute of Health and Medical Research (INSERM)
Liebaers, Inge	41	3119	41	Free University of Brussels
Remohi, Jose	43	3061	43	University of Valencia
Meseguer, Marcos	40	2829	36	University of Valencia
Ferraretti, Anna P.	22	2466	20	SISMeRReprod Med Unit, Bologna
Pellicer, Antonio	46	2464	40	University of Valencia
Camus, Michel	32	2435	32	Free University of Brussels
De Sutter, Petra	69	2426	60	Ghent University
Tesarik, Jan	29	2374	26	ClinicaMargen Infertility clinic in Granada
Goossens, V.	12	2283	12	ESHRE Central Office
Greco, Ermanno	37	2267	34	Center for Reproductive Medicine, Villa Mafalda, Rome,

TLS, total link strength.

### Top cited articles

**Figure 5:**
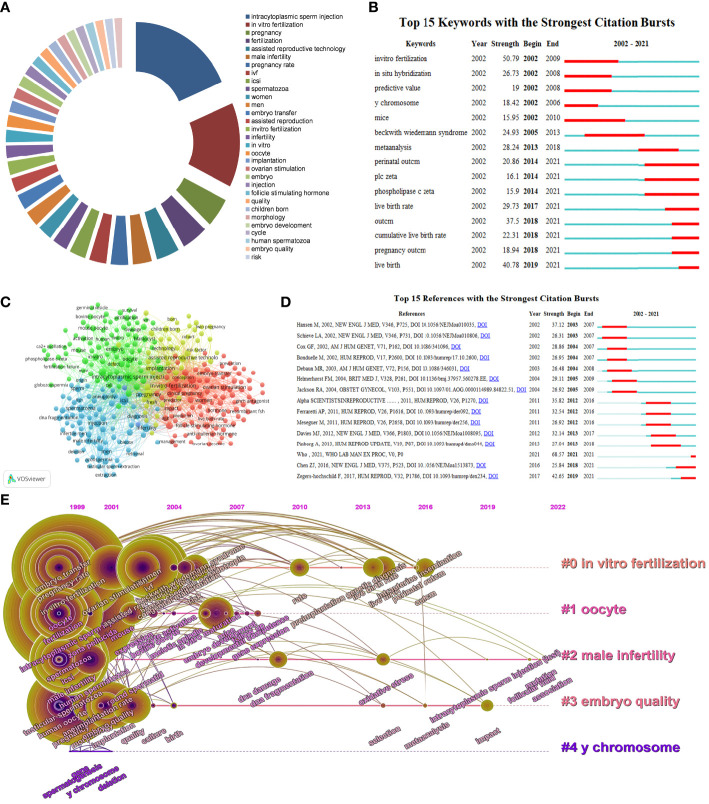
Visual cluster analysis of co-occurrence among keywords of ICSI. Top keywords in terms of their frequency in ICSI research **(A)**.Top 15 keywords with the strongest citation bursts from 2002 to 2021. The red bar means that researchers in that time highly emphasize the keyword **(B)**. The frequency of occurrence and co-occurrence were displayed by VOSviewer **(C)**. The strongest citations bursts of references in different stages were concluded by CiteSpace **(D)**. The strength value indicates the degree of a burst **(B, D)**. Keywords were grouped in five clusters, timeline distribution of the top 5 clusters and keywords trends by year were visualized from CiteSpace **(E)**.

The most cited articles are usually considered landmarks because of their referential value. The co-citation analysis revealed that 5932 publications were cited more than ten times. The online database, WoSCC, revealed that 605 publications were cited more than 100 times. We concluded on the top 15 cited articles which are related to ICSI items (**Table 1**). The most cited publication was *“American Society of Clinical Oncology recommendations on fertility preservation in cancer patients.”* In this paper, Lee et al. mentioned the topic of fertility preservation and talked about how to choose an appropriate plan to preserve fertility in cancer patients. They recommended that sperm and embryo cryopreservation are considered standard practice and were widely available for reproduction *via* ICSI (17). These highly-cited publications could provide a reliable reference for researchers, and ICSI had an important role in the fields of reproductive medicine. Four out of 15 publications, which concentrated on fertility preservation, were cited at a high frequency. In addition, the risk of birth defects after ICSI was a hotly-debated subject. Six studies were highly cited among the top 15 publications. The results indicated that researchers paid close attention to fertility preservation and the risks of birth defects during the past 20 years. The landmark articles and coocurrence on ICSI research can be found in **Figure 4D**. To track the concentration in different stages, the top 15 references with strongest citation bursts was visualized in **Figure 5D**.

### Top contributing and core journals

The numbers of publications and citations indicate the impact of a journal in a given research field. The top 15 most productive journals published 4702 (56.85%) manuscripts on ICSI between 2002 and 2021 as shown in **Table 6**, alongside their 2021 impact factor and JCR category. Of these journals, *Human Reproduction* and *Fertility and Sterility* published the most papers on ICSI research (1051/8271, 12.71% and 1046/8271, 12.65%, respectively), and they were the most cited journals. The results suggested these two journals were the core journals of the field of ICSI research. Most of the top publishing journals were categorized as reproductive biology, obstetrics & gynecology, and andrology journals (**Figure 4C**).

**Table 6:** Top 15 most cited journals in ICSI research.

Journal	Documents	Citations	TLS	2021 IF	JCR Category
Human Reproduction	1051	57118	12213	6.353	Obstetrics & Gynecology - Scie(Q1); Reproductive Biology - Scie(Q1)
Fertility And Sterility	1046	47414	11378	7.49	Reproductive Biology - Scie(Q1)
Reproductive Biomedicine Online	623	18814	6723	4.567	Reproductive Biology - Scie(Q1); Obstetrics & Gynecology - Scie(Q1)
Journal Of Assisted Reproduction And Genetics	596	8916	4737	3.357	Obstetrics & Gynecology - Scie(Q2); Genetics & Heredity - Scie(Q3); Reproductive Biology - Scie(Q3)
Gynecological Endocrinology	187	1738	992	2.277	Obstetrics & Gynecology - Scie(Q3); Endocrinology & Metabolism - Scie(Q4)
Andrologia	181	2078	1341	2.532	Andrology - Scie(Q3)
European Journal Of Obstetrics & Gynecology And Reproductive Biology	151	2621	1042	2.831	Reproductive Biology - Scie(Q3); Obstetrics & Gynecology - Scie(Q3)
Theriogenology	147	3532	1182	2.923	Veterinary Sciences - Scie(Q1); Reproductive Biology - Scie(Q3)
Reproductive Biology And Endocrinology	130	2467	1203	4.982	Reproductive Biology - Scie(Q1); Endocrinology & Metabolism - Scie(Q2)
Biology Of Reproduction	129	5702	1144	4.161	Reproductive Biology - Scie(Q2)
Archives Of Gynecology And Obstetrics	112	1319	804	2.493	Obstetrics & Gynecology - Scie(Q3)
Zygote	96	794	712	1.818	Reproductive Biology - Scie(Q4); Cell Biology - Scie(Q4); Developmental Biology - Scie(Q4)
Clinical And Experimental Obstetrics & Gynecology	89	243	359	0.192	Obstetrics & Gynecology - Scie(Q4)
Frontiers In Endocrinology	86	484	595	6.055	Endocrinology & Metabolism - Scie(Q1)
Andrology	78	941	823	4.456	Andrology - Scie(Q1)

TLS, total link strength; IF, impact factor; JCR; journal citation of reports.

### Mapping and analysis of keywords

The highest frequency of keywords can be mapped based on their frequency of occurrence, and keywords were classified based on their number of appearances by VOSviewer (**Figure 5A**). A keyword analysis is used to identify current hotspots and get insight into the overview of a field; moreover, it can also determine the trends and future directions (18). To determine the evolution of the research hotspots, the strongest citation bursts of keywords were illustrated by CiteSpace in **Figure 5B**. The outcome of the pregnancy and the live birth rate have appeared consistently from 2014 to 2021, indicating that more and more studies have focused on improving embryonic quality and newborn health since 2014. **Figures 5C, E** show the co-occurrence analysis of the most frequent keywords. The different colors represented the clusters revealed by the VOSviewer analysis. The size of the node represents the frequency use of the keyword, and the thickness of the connecting line represented the strength of the relationship between these keywords. According to the frequency of occurrence, the pregnancy rate and male infertility were constant foci of attention. In the past two decades, ICSI, as an important component of ART, had been greatly promoted and clinically applied. However, the development characteristics are different in certain stages. More studies have focused on embryonic quality and reproductive health in recent years. With the deepening understanding of this technology, ICSI has become an effective weapon against male infertility. However, we need to pay more attention to the risks of live birth.

### Research shared between ICSI and COVID-19

In late 2019, the first case of Coronavirus disease 2019 (COVID-19) was reported in Wuhan, China. The World Health Organization (WHO) declared the outbreak of the epidemic an international emergency on January 30, 2020 (19). The rapid human-to-human transmission of the disease *via* direct contact or droplets has been confirmed. The epidemic had many negative impacts on medical systems. Especially, the suspicious sequelae of those severe cases should be followed up carefully for a long time. To know if COVID-19 could have disadvantages on the outcomes of ICSI or ART, we extracted relevant references from the WoSCC database by using the search terms “ICSI” and “COVID” to explore the database from 2019 to 2021. There were only nine publications that focused on the relationship between ICSI and COVID-19. The major topics have been observing whether COVID-19 would affect the number of fertility consultations, successful pregnancy rates, and protective measures from the virus’ hazard throughout the medical procedure. The overall results show that COVID-19 had minimal impact on ICSI outcomes and no negative impact on implantation rates or on live births (**Table 7**). The quality of the patient’s semen and embryos did not decrease due to COVID-19 (20–22). More data and longer follow-ups are needed to validate the previous conclusions.

**Table 7:** The research between ICSI and COVID-19.

First Author	Article Title	Journal	Year	Corresponding Orgnazation
Jacobs, Emily;	Fresh Embryo Transfer Cycle Characteristics and Outcomes Following *In Vitro* Fertilization *via* Intracytoplasmic Sperm Injection Among Patients With and Without COVID-19 Vaccination	Jama Network Open	2021	University of Iowa
Kolanska, Kamila	Mild COVID-19 infection does not alter the ovarian reserve in women treated with ART	Reproductive Biomedicine Online	2021	INSERM
Alviggi, Carlo	COVID-19 and assisted reproductive technology services: repercussions for patients and proposal for individualized clinical management	Reproductive Biology and Endocrinology	2020	University of Naples Federico II
Li, Fei	Controlled Ovarian Hyperstimulation Protocol in Infertile Patients During the COVID-19 Pandemic	Frontiers in Physiology	2021	Zhengzhou University
Cutting, Elizabeth	The impact of COVID-19 mitigation measures on fertility patients and clinics around the world	Reproductive Biomedicine Online	2021	Monash University
Rajput, Sandeep K	Absence of SARS-CoV-2 (COVID-19 virus) within the IVF laboratory using strict patient screening and safety criteria	Reproductive Biomedicine Online	2021	CCRM Fertility Network, Lone Tree CO.
Wang, Meng	Investigating the impact of SARS-CoV-2 infection on basic semen parameters and *in vitro* fertilization/intracytoplasmic sperm injection outcomes: a retrospective cohort study	Reproductive Biology and Endocrinology	2021	Huazhong University of Science and Technology
Porcu, Eleonora	High-security closed devices are efficient and safe to protect human oocytes from potential risk of viral contamination during vitrification and storage especially in the COVID-19 pandemic	Journal of Assisted Reproduction and Genetics	2021	University of Bologna
Porcu, Eleonora	Successful Pregnancies, Births, and Children Development Following Oocyte Cryostorage in Female Cancer Patients During 25 Years of Fertility Preservation	Cancers	2021	University of Bologna

### Latest reviews in the field of ICSI research

Literature reviews are comprehensive summaries of previous research on a specific topic. They are essential to learning current research topics and are the keystone of evidence-based medicine. A literature review is able to summarize and synthesize the existing scholarly research on a particular topic. It critically analyses the information gathered by identifying areas that require further research and reviewing areas of controversy. The following data included the top 15 most cited reviews on ICSI during the past 12 months (**Table 8**). The *‘Human Reproduction Update’* journal was the most prolific source of reviews on ICSI. Four main subfields were focused on: sperm quality and male infertility, dosage regimens of ovarian stimulation, preferential oocyte and embryo quality, and updated guidelines on the diagnosis and management of infertility. Six publications were assigned to the subfield “sperm quality and male infertility.” The results suggested that ICSI remains a crucial treatment for male infertility. Furthermore, how to improve the quality of the sperm, oocyte, and embryo is still a hotspot in the field of ICSI research.

**Table 8:** Top 15 high-cited reviews in ICSI research during the latest 12 months .

Author	Article Title	Journal	Citations
Agarwal, Ashok	Male infertility	Lancet	150
Carson, Sandra Ann	Diagnosis and Management of Infertility A Review	Jama	49
Zaat, Tjitske	Fresh versus frozen embryo transfers in assisted reproduction (Review)	Cochrane Database of Systematic Reviews	26
Nirgianakis, Konstantinos	Fertility, pregnancy and neonatal outcomes of patients with adenomyosis: a systematic review and meta-analysis	Reproductive Biomedicine Online	20
Datta, Adrija Kumar	Mild versus conventional ovarian stimulation for IVF in poor, normal and hyper-responders: a systematic review and meta-analysis	Human Reproduction Update	18
Ribas-Maynou, Jordi	Clinical implications of sperm DNA damage in IVF and ICSI: updated systematic review and meta-analysis	Biological Reviews	18
Sang, Qing	Genetic factors as potential molecular markers of human oocyte and embryo quality	Journal of Assisted Reproduction and Genetics	17
Nikshad, Aylin	Advances of microfluidic technology in reproductive biology	Life Sciences	14
Gualtieri, Roberto	Sperm Oxidative Stress during *In Vitro* Manipulation and Its Effects on Sperm Function and Embryo Development	Antioxidants	12
Coticchio, Giovanni	Plasticity of the human preimplantation embryo: developmental dogmas, variations on themes and self-correction	Human Reproduction Update	12
Capalbo, Antonio	Preconception genome medicine: current state and future perspectives to improve infertility diagnosis and reproductive and health outcomes based on individual genomic data	Human Reproduction Update	12
Allen, Christopher P.	Outcomes of pregnancies using donor sperm compared with those using partner sperm: systematic review and meta-analysis	Human Reproduction Update	12
Andrade, Danilo L.	Differential Diagnosis of Azoospermia in Men with Infertility	Journal of Clinical Medicine	11
Diaz-Hernandez, Indra	Uterine natural killer cells: from foe to friend in reproduction	Human Reproduction Update	10
Dai, Changsheng	Advances in sperm analysis: techniques, discoveries and applications	Nature Reviews Urology	9

## Discussion

The increase in the prevalence of infertility is a serious health problem. There are about 50 million couples experiencing fertility-related issues worldwide, and 30%-50% of them are caused by male factors (23, 24). The reductions in male sperm concentration, motility, and morphology, which have occurred over the past few years, were related to environmental pollution, obesity, unhealthy diet, and work stress. Women’s ovarian reserves are also declining due to work stress, delayed childbearing, and so on (25). The frequency of use of ART has been increasing over the past two decades, with more than 2.5 million cycles being operated every year (26). During the ART cycle, a selected subpopulation of 25,000–150,000 sperms is placed around an oocyte to initiate fertilization in IVF. Unlike IVF, ICSI requires more careful manipulation of a single sperm that is injected into an oocyte in the laboratory. ICSI was introduced as an efficient form of ART in 1992 for the treatment of male infertility. With ICSI, Men with severe oligozoospermia or azoospermia have the opportunity to produce genetically-linked offspring due to ICSI (27). The percentage of IVF cycles with the use of ICSI increased dramatically from 11.0% in 1995 to 57.5% in 2004 in the United States, while the percentage of infertility attributed to male factors remained constant (28).

The present bibliometric analysis explored the contributions, impact, research hotspots, and trends in the ICSI field during the last two decades. The main research areas, according to the WoSCC database, were ‘Reproductive Biology’, ‘Obstetrics and Gynecology’, ‘Genetics Heredity’ and ‘Andrology’. The number of publications increased quickly from 2018 onward, suggesting a generally increasing interest in research on ICSI area. However, the present study has some limitations. Firstly, similar to other bibliometric analyses, the documents were extracted only from the WoSCC database because of the rules of the system algorithm. Secondly, the authors’ addresses were not written normatively, certain institutions’ contributions may have been dispersive, and the rankings were influenced slightly. Lastly, the synonyms of keywords could affect the emergence analysis of keywords, although we tried to remove these heterogeneities as much as possible to reduce the resultant bias.

The United States, Belgium, Japan, Italy, and the UK contributed the majority of publications and were responsible for the highest quote rates in ICSI studies from 2002 to 2011. After 2018, the number of publications from Chinese institutions increased rapidly, with publications from Chinese academic institutions accounting for 32.10% of the total number of publications in the field of ICSI research from 2018 to 2021. By contrast, the number of American studies has remained fairly constant in the past 20 years. However, the number of Chinese publications increased exponentially from 5 in 2002 to 208 in 2021. The most influential institution in the field of ICSI research was the Free University of Brussels.

*Human Reproduction* and *Fertility and Sterility* were the core sources of publications. These two journals had the most citations and publications in ICSI research. The top three most cited papers were “American Society of Clinical Oncology recommendations on fertility preservation in cancer patients,” “Highly efficient vitrification method for cryopreservation of human oocytes,” and “Perinatal outcomes in singletons following *in vitro* fertilization: A meta-analysis.” The results indicated that researchers focused a lot on the preservation of oocytes, sperms, or embryos during the past 20 years. The outcomes and risks of pregnancy always drew the attention of clinicians. These topics will continue to be debated in the next few years.

The latest reviews were extracted for tracking the focused issues of ICSI between 2021 and 2022. *Human Reproduction Update’* had the highest number of ICSI reviews. These four subfields were focused on: sperm quality and male infertility, dosage regimens of ovarian stimulation, preferential oocyte and embryo quality, and updated guidelines for the diagnosis and management of infertility. After that, the authors searched all publications that focused on the relationship between ICSI and COVID-19, and there were only nine of such publications. The results of these highly-cited articles suggested that COVID-19 has neither a substantial influence on the outcomes of ICSI nor an effect on infertile couples’ consultation for now.

As the indications have expanded and the number of cycles of ICSI has increased, more attention has been focused on the safety and stability of this technology. As of 2020, an estimated 8 million children had been conceived *via* ART. The American Society of Reproductive Medicine declared ICSI might be a safe and effective technology for the management of male infertility in 1994. Generally, ICSI is considered as a safe alternative for couples who would be unable to achieve a successful pregnancy (29, 30). There were potential controversies regarding offspring obtained *via* ICSI in the following aspects: the obstetrical outcomes of pregnancies, chromosomal abnormalities associated with the offspring obtained *via* ICSI, congenital malformations of the newborns resulting from ICSI procedures, and developmental abnormalities in postnatal children resulting from ICSI (29). With the increased incidence of preterm birth and low birth weight associated with ART, there have been concerns about postnatal growth. Theoretically, there are potential risks from ICSI manipulation. The injection of the oocyte might cause damage to the ooplasm or meiotic spindle apparatus. The external substances and contaminants could affect the microenvironment of the oocyte. The male gamete might also bring hazards because of potentially risky sperm, which include structural defects, Y-chromosome deletions, DNA fragments or breaks, and aneuploidy that could be injected into a healthy oocyte. The process of genomic imprinting might be changed during gametogenesis or maintained incompletely during embryonic development (31). The outcome data of childhood and growth into early adulthood are the potential hotspots and trends of ICSI. Continuous follow-up into later life needs to be investigated and confirmed (32). Recent studies suggested a small excess of birth defects and low birth weight in children conceived *via* ART. Furthermore, several studies have reported an increased frequency of ART conceptions associated with Beckwith–Wiedemann syndrome or Angelman syndrome caused by an imprinting defect (32, 33). The keywords with the strongest citation burst result indicated the focus and hotspots of ICSI research, concentrating on the outcomes of pregnancy and risks of live birth from 2014 onward. In addition, the clinical pregnancy rate and outcomes of frozen–thawed embryo transfer or fresh embryo transfer and how to improve the quality of semen for ICSI, are hotspots and issues from keyword analysis results. Nowadays, ICSI is a frequently procedure applied worldwide to treat infertile couples. As an invasive method, ICSI surpasses all the process of physiological events in normal fertilization, thus is frequently debated as potentially increasing risk in future generations. Despite the above observations, the evidence concerning an increased risk of diseases in ICSI offspring remains confusing. It’s always worth to keep an eye on this field. More studies are needed to evaluate the safety of ICSI on long-term health and developmental outcomes in offspring.

## Conclusion

To the authors’ knowledge, this is the first study to assess the ICSI research status *via* a bibliometric analysis. In this study, a systematic view of ICSI research hotspots, future directions, most cited authors, journals, and institutions was conducted using CiteSpace or VOSviewer. The results would help researchers to better understand the current situation, trends and hotspots, and future development of this field. European institutions are the most impactful in the world regarding the study of ICSI. Research hotspot analyses suggested that male fertility, fertility preservation and risks of live birth remain major concerns in the next few years. International collaboration, which helps researchers to easily keep up with academic progress, was common in this area.

## Data availability statement

The original contributions presented in the study are included in the article/Supplementary Material. Further inquiries can be directed to the corresponding authors.

## Author contributions

YZ and GH designed the study. WH, TX, and HY searched the database. XS, TX, and GH analyzed the data. Manuscript was written by XS. and GH. All authors contributed to the article and approved the submitted version.
